# The embedded research model: an answer to the research and evaluation needs of community service organizations?

**DOI:** 10.1186/s12961-024-01246-z

**Published:** 2024-11-20

**Authors:** Bianca E. Kavanagh, Vincent L. Versace, Kevin P. Mc Namara

**Affiliations:** 1 Deakin Rural Health, School of Medicine, Deakin University, Princes Highway, Warrnambool, VIC 3280 Australia; 2 Brophy Family and Youth Services, Warrnambool, Australia

**Keywords:** Quality improvement, Health services, Community services, Australia, Research capacity building

## Abstract

There is an increasing need to provide evidence of outcomes within the community services sector. However, funding challenges, workforce pressures, and the complex social contexts in which community service organizations operate limit their potential for organizational capacity building. This has flow on effects on the ability to conduct impactful and strategic research. The embedded research model (i.e., when a researcher is embedded into a “host” organisation) may aid in building research and evaluation capacity. This may be particularly useful for the purposes of routine outcome monitoring and continuous quality improvement, which may in turn lead to opportunities for local research and evaluation through the embedded research model. Previous research on the embedded research model across various settings has suggested a number of lessons for implementation. However, to date, nil research has focused on community service organizations. Additional considerations need to be made within this context due to resource limitations, ethical issues, and diverse reporting requirements. Such considerations include the need to take a ‘slow science’ approach to research and evaluation outputs, consideration of the organisation’s readiness for change, and the need to report activities in a transparent, thorough, and consistent manner. The endorsement of embedded research in community service organisations may aid in providing evidence of outcomes for clients, and simultaneously, increase the ability for policymakers to evidence-informed decisions on how to improve outcomes for the local population.

Across the globe, community-based health and social services provide support to sustain and foster the functioning of individuals, families, and groups to augment their potential and improve community wellbeing [1, 2]. Although their delivery and funding differs across the international context [3], the decisions and implementation of community-based health and social services lay at the community level, either with local government or civil society[2]. Within the Australian setting, community services organizations (CSOs) sit within this sector and are the not-for-profit and non-governmental organizations that are contracted by the government to deliver certain social services by providing support and material assistance to the public [4].

CSOs often have multiple funding avenues—spanning philanthropic, and state and federal government sources—and are delivered within complex and often time-limited contexts. CSOs face increasing workforce pressures due to less than full-cost funding of services, demand for services outweighing supply [5] and the impact of contract-to-contract funding undermining workforce development [6]. These challenges limit the potential of capacity building (i.e., the planned and system-wide effort to enhance organizational performance via purposeful reflection, planning, and action, and the development of skills and resources to achieve organizational goals) within CSOs [6]. As a result of the limited analytical resources to conduct strategic research, it is difficult for CSOs to capture, analyse, and report evidence of outcomes [6], despite many funding opportunities being contingent on doing so. One way to increase the evidence of outcomes in CSOs is to utilize the embedded research (ER) model (i.e., when a researcher is embedded into a “host” organisation) to build staff capacity to conduct research and evaluation.

Various versions of the ER model have been demonstrated in healthcare and other settings in recent decades. A review by Vindrola-Padros et al. [7] identified eight healthcare-related and nine non-healthcare related (i.e., education, judicial system, social care, science policy) publications on ER approaches. Although nil articles in the review were specific to CSOs, it was identified that the ER model may be pertinent to social care settings where there is strong evidence for a specific practice or where practice tools can be easily customized to the local context [8]. This model may be applicable to CSOs, which often provide tailored services and practice approaches that are guided by the local context. The consideration of the local context may be even more relevant to CSOs that operate with a place-based approach—particularly those in regional, rural, and remote settings.

Although, to the authors’ knowledge, there are nil studies that describe the ER model with the Australian CSO context, Vindrola-Padros et al. [7] posit that there are shared features across different settings which exemplify the ER model. These include that the researcher: (1) is generally affiliated with both an academic institution and an organisation that is separate from academia, therefore working in a position of “in-between-ness”; (2) develops relationships with staff and is viewed as a member of the team; (3) produces knowledge with local teams (co-produced) that responds to the host organization’s needs; and (4) builds research capacity in the host organization. Further, the ER model is uniquely able to sit in the “middle ground” between research and evaluation [9], which is critical to knowledge generation and capacity building.

Recently, the Productivity Commission identified that within the Australian healthcare system, local level evaluation is inconsistent, there is insufficient activity focused on reducing waste, and that in-house evaluation capacity needs to be increased [10]. Although CSOs operate outside of the healthcare system, the nature and challenges of the community service setting likely further increase the issues of conducting local level evaluation for CSOs. This is reinforced by the notion that presently, there is no agreed measurement and evaluation framework for not-for-profits. The Productivity Commission posited that such a framework would be useful to improve the rigour and consistency of evaluation results, improve reporting requirements and provide greater understanding the sector’s role in the community [5]. This is critically important, as the absence of evidence produced by CSOs likely reduces the ability to make evidence-informed decisions to improve outcomes and processes, and to provide evidence for funding opportunities, particularly for context-dependent care [11].

One way to increase the opportunity for local evaluation is through routine outcome monitoring and continuous quality improvement [12]. These approaches can facilitate immediate modification to service delivery via feedback loops, through the continual gathering and implementation of learnings derived from local data [13]. Carey suggests—within the context of rural and remote health services—that in order for continuous quality improvement to be effective, practitioners and evaluators need to work in close collaboration within a working alliance [13]. This working alliance includes a willingness to be visible, open to learning and change, and to work in the context of safety and trust [13]. Further, for ongoing routine outcome monitoring and evaluation to take place, organizations need to have engaged people who are dedicated to the value of the work, and have processes that are responsive to varying circumstances and conditions, which are reinforced by accessible resources [13]. Notably, Carey’s research emphasized the need for external evaluators to conduct independent evaluations. This was viewed as important as external evaluators can be a “naïve observer”, with the ability to hold programmes to account in a way that may be difficult for an internal member of the organization to enact [13]. The ER model may thus facilitate the ability for embedded researchers in CSOs to conduct ongoing monitoring and evaluation through their familiarity with the host organisation and, simultaneously, an ability to maintain a degree of distance to enable accountability.

Given the challenges for CSOs to conduct research and evaluation, and simultaneously the potential for ER models to address such challenges, a unique opportunity exists to utilize the ER model in CSOs. However, considering the paucity of peer-reviewed literature in this context, the approaches to implementation and structure of the model remains unclear. Vindrola-Padros et al. [7] provided several lessons for embedded researchers based on their review findings. These include (1) the experiences and viewpoints of various subgroups within the host organisation need to be considered, (2) the development of collaborative relationships between staff in the host organisation and the co-production of knowledge can be bolstered through reflexivity, (3) clear guidelines should be agreed upon from the outset to manage expectations, (4) regular meetings should be held with relevant teams and groups to offer iterative feedback, and (5) embedded researchers should maintain links to their academic institutions to maintain a critical perspective [7].

Other lessons learned from ER initiatives in other settings include that the immersion of the researcher within the host organisation permits the knowledge of relevant managers with decision-making powers to flag issues, develop linkages, and enable change [14]. A willingness from leadership to participate in research initiatives and a drive to lead quality improvements have also been emphasized as being critical to creating a learning culture [15] and for utilizing research [16]. Further, novel approaches by managers are needed to allow knowledge co-production [17]. This includes a willingness from managers to trust and permit researchers to participate in the managerial terrain. Tensions from both the host organization and embedded researcher may arise through this process. This may occur as the formal mandates and informal practices of management approaches may be questioned or shaped by the embedded researcher; thus, management must have capacity to shift from a reactive response to proactive planning and research design [17]. The introduction of an embedded researcher may indicate a desired organizational or cultural change on the part of senior management, as well as the need for new skillsets and approaches to enable this. This may contest the self-identity of some managers and promote a resistive response, depending on how the overall change is perceived. In addition, patience may be required as research tends to operate more slowly than regular management timelines [17].

At Brophy Family and Youth Services (Brophy; where the first author works as an embedded researcher), we have spent the previous 2 years implementing the ER model and have begun piloting routine outcome monitoring in several programmes. Based on our experience to date, we agree with Vindrola-Padros et al. [7] assertions but also recommend additional principles to be employed for ER models specifically within CSOs. Firstly, embedding research in CSOs should take a “slow science” approach [18], allowing the organization and embedded researcher to grow together. The slow science approach signifies that research is acutely intertwined with wider social interests and that research must be undertaken with those who will use and benefit from it [18]. Further, this approach may allow the latent benefits of research—such as collective learning and confidence to learn—to be explored in areas of the organization, which may not see the direct value of research [19]. For embedded researchers in CSOs, this may mean a focus on co-producing quality research over the long term, with recognition that results (and outputs) may occur after several years. It is acknowledged that the slow science approach may be at odds with traditional output-focused research, which is often practised within discrete projects over short timeframes and against the constraints of funding cycles [20]. Further, consideration of the readiness for change (i.e., to implement research and evaluation activities within the CSO) should be undertaken at the beginning to understand leadership engagement, available resources, and access to knowledge and information [21]. Assessment of readiness is critical to understanding the current landscape and to gauge exposure and interest in conducting research and evaluation in place-based organisations [22]. This may be relevant to CSOs, whereby the assessment of readiness may indicate where the organisation sits within its journey to continuous quality improvement, providing an insight into where an embedded researcher may focus their efforts to have the greatest impact.

In summary, the community services sector may benefit from using the ER model, particularly if the aforementioned learnings from its utilization in the health service sector is applied. These learnings also include that that ER activities are reported in a transparent, detailed, and consistent manner to provide evidence of a descriptive and evaluative appraisal of ER initiatives [23]. Reporting may be evidenced through the development of a checklist to standardize and consider the crucial factors, tasks, or criteria of ER activities. Checklists have been shown to improve uptake and implementation efficiency in research implementation environments [24]. Although such checklists may need to be developed in a bespoke manner relevant to the particular ER initiative, Prausnitz et al. [24] suggest that such checklists should include brief information about the purpose, questions to guide stakeholder engagement, and transition-to-implementation goals and timeline. The importance of reporting is also echoed by Carey, who suggested that failing to publish precludes the generation of a body of evidence concerning the effective implementation and sustainability of continuous quality improvement [13]. Further, and in addition to the advantage of generating purposeful research with and for those who will benefit from it, the ER model proposes several benefits within the community service sector. These benefits are economic—as research and evaluation activities do not need to be outsourced and are an investment in the local community—as by virtue, the “embedding” of researchers must happen at the local level; social and health—as results generated through ER activities are directly applied to and with the clients the CSO services, and mutual partnership between the host organisation and the academic institution. The ER model also allows embedded researchers to be familiar with the context of the host organisation, while facilitating some level of separation for accountability.

Notwithstanding the benefits of the ER model in the community service sector, there are undoubtedly challenges to be faced. These challenges include resource limitations, including competing time demands for CSO staff to conduct research and evaluation activities [5], ethical considerations for the embedded researcher [25], and issues with data collection methods due to system limitations and diverse reporting requirements for differently funded programmes [5]. Further, tensions and a sense of culture shock may arise for the embedded researcher, including the consideration of how to manage boundaries and conflicts of interest, as well as how to articulate and consider the scope of research design options available [26]. Such tensions may result in the varying and often unexamined expectations between the host organization and embedded researcher; the complexity in evaluating and showing the value of research initiatives, the necessity to act on staff changes and external influences and the intricacy in reconciling divergent elements of multiple initiatives [26–28]. Nonetheless, the benefits of the ER model in supporting CSOs to conduct research and evaluation activities appear to far outweigh such challenges, and uptake of the model should be considered in CSOs that are required or want to evidence outcomes for clients.

## Data Availability

No datasets were generated or analysed during the current study.

## Abbreviations

CSOs: Community service organisations
ER: Embedded research
